# Degradation Mechanism of Ag Nanorods for Surface Enhanced Raman Spectroscopy

**DOI:** 10.1038/s41598-017-16580-2

**Published:** 2017-11-24

**Authors:** Lou Bachenheimer, Ryan Scherzer, Paul Elliott, Stephen Stagon, Lev Gasparov, Hanchen Huang

**Affiliations:** 1 Department of Mechanical Engineering, University of Connecticut, Storrs, CT 06269 USA; 2 Department of Mechanical Engineering, University of North Florida, Jacksonville, FL 32224 USA; 3 Department of Mechanical & Industrial Engineering, Northeastern University, Boston, MA 02115 USA; 4 Department of Physics, University of North Florida, Jacksonville, FL 32224 USA

## Abstract

This paper reports a degradation mechanism of silver (Ag) nanorods that are used as substrates for surface enhanced Raman spectroscopy (SERS). The attachment of sulfur and hydrocarbons to the surfaces of Ag nanorods is observed when they are stored in ambient over four months. This attachment is observed to correlate with ~20% decrease in SERS signal. The attachment, and thereby the signal degradation, takes three weeks to complete, and remains stable after the initial decay over the rest of the four month test period. While this degradation mechanism is a limitation to the gross enhancement, the ensuing stability beyond three weeks is encouraging.

## Introduction

For surface enhanced Raman spectroscopy (SERS) applications^1–3^, Ag nanorods from physical vapor deposition (PVD) have two major advantages over nanomaterials from alternative synthesis methods: one may tailor nanorod morphologies to maximize SERS sensitivity for specific applications, and clean nanorod surfaces lead to excellent SERS sensitivity^1–3^. To maximize the benefit of these two advantages, it is critical that the Ag nanrods do not degrade substantially during their course of use.

The morphological features of Ag nanorods that lead to large SERS enhancement include small diameters and small, but clear, separations between nanorods^2,3^. However, these features also lead to their susceptibility to degradation at the elevated temperatures required by some SERS applications^2^. Even at about 50 °C, just above ambient, substantial coarsening of Ag nanorods takes place in a matter of minutes in air when their diameters are small and they are separated^4–6^. As a result of the coarsening, SERS sensitivity decreases^2,5^. To minimize the coarsening, capping or coating with a high melting temperature material has been introduced to either slow down the surface diffusion or to mitigate the mass transport effects of the surface diffusion of Ag nanorods^2,5,7,8^. While capping works to maintain the signal under harsh coniditions, it also causes a reduction in signal strength.

While the Ag nanorods have clean surfaces immediately after fabrication, the chemical inertness of Ag becomes questionable at the nanoscale. It has been reported that Sulfur (S) atoms attach easily to Ag nanoparticles^9^. For applications of Ag nanorods, the ability for the target molecule to attach directly to the Ag surface is paramount to SERS sensitivity. Based on the literature report, similarly to Ag nanoparticles^9^, we expect that sulfur atoms will attach to Ag nanorods. However, it is unclear (1) whether the S atoms will remain attached to Ag nanorods for an extended period of time as morphologies and high energy binding locations may change or disappear due to gradual surface energy minimization, and (2) how the attachment of S atoms will affect the SERS sensitivity of Ag nanorods. The reduction of high energy binding locations derives from natural energy minimization of surfaces as diffusion takes place.

This paper reports the experimental results that (1) S and hydrocarbons attach to Ag nanorods within four weeks (28 days) but they do not attach to more inert gold (Au) nanorods; and (2) the attachment of S and hydrocarbons reduces the SERS signal of the Ag nanorods by about 20%, which then remains stable for four months after the initial attachment.

## Results

**Figure 1:**
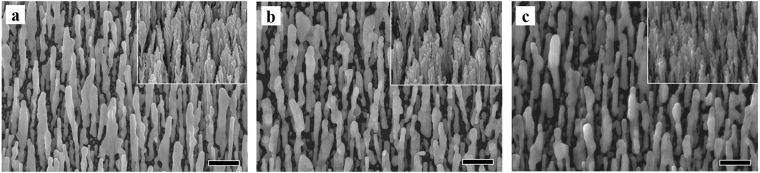
SEM images of Ag nanorods, (**a**) as deposited, (**b**) 4 weeks (28 days) later, and (**c**) 4 months (115 days) later; with the insets showing analogous Au nanorods. The scale bar is 500 nm for all the images and insets.

As the first set of experimental results, Fig. 1 shows that the morphology of Ag nanorods does not substantially change with time. The average measured diameters are 118 nm on day 1, with a standard deviation of 35 nm (30%), 113 nm on day 28, with a standard deviation of 40 nm (35%), and 131 nm on day 115, with a standard deviation of 43 nm (33%). The difference in average diameter from day 1 to day 28 is less than 5%, and is well within the natural spread of nanorod diameters, as seen by the large standard deviations. For comparison, using day 1 as a baseline, the average change in diameter on day 28 is −5% and on day 115 is 11%. The data also shows that the nanorods at both extremes, smallest and largest, are within the same range for all three measured cases and range from ~75 nm to ~200 nm. It is also noted that the center to center spacing of adjacent nanorods remains nearly unchanged over time, with a measured average of ~200 nm for all three cases. It is noted that a contributor to the visual difference between day 1 and day 28 is the absence of the very small particles in between nanorods by day 28, which is expected due to normal diffusivity. Scanning electron microscope (SEM) images of analogous Au nanorods, which are expected to be inert, are included as insets to Fig. 1. Unlike the smooth surfaces of Ag nanorods, the Au nanorods grown here demonstrate branching and bundling, and a numerical analysis is not applicable. However, no morphological change is observable over this time period for the case of Au.

**Figure 2:**
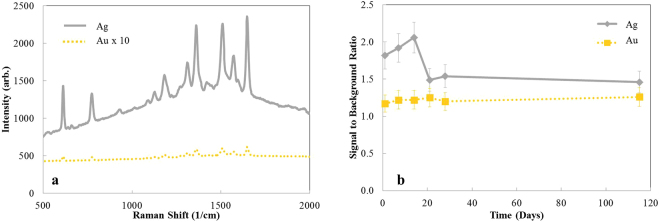
(**a**) SERS signal recorded for spectra of aqueous 1 × 10^−6^ M Rhodamine 6 G (R6G) on Ag and Au nanorods in Fig. 1a, and (**b**) a characteristic SERS signal-to-background-ratio of Ag and Au nanorods as a function of time.

Moving beyond the change in morphology, Fig. 2a shows the SERS spectra of 1 × 10^−6^ M Rhodamine 6 G (R6G) absorbed onto the Ag and Au nanorods that are shown in Fig. 1a. It is clear that the SERS sensitivity of Ag nanorods is much higher than that of Au nanorods. As a numerical measure, we use the peak intensity at 1647 cm^−1^ to determine the signal-to-background ratio. Figure 2b shows a substantial drop of SERS signal-to-background-ratio of Ag nanorods after three weeks (21 days), by roughly 20%. Further, this reduction remains fairly constant for a duration of four months (115 days). In comparison, no such change is observed for Au, which remains nearly constant over the entire four month time period. As there is no significant change in morphology from day 1 to day 28, we propose that surface chemical changes must be responsible for this change in intensity observed only for Ag. Surface contamination of nanorods have been shown to reduce the SERS effectiveness^10^.

**Figure 3:**
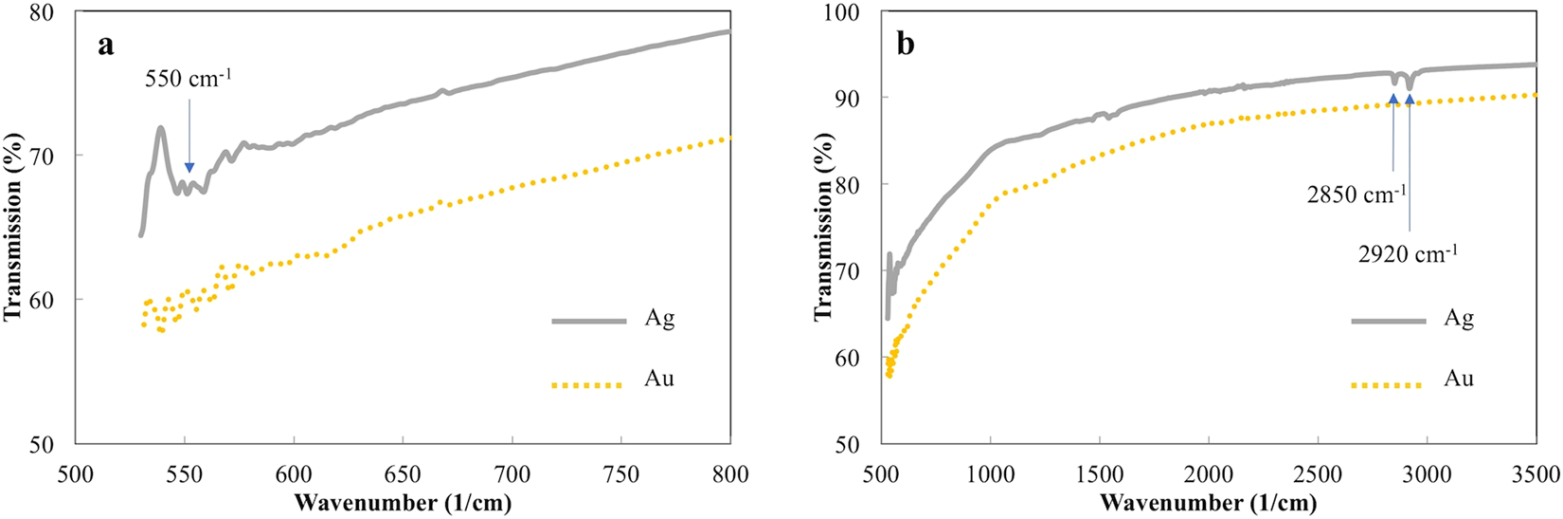
(**a**) FTIR of Ag and Au nanorods after 28 days, with (**b**) showing the spectra over a larger wavenumber range.

To understand the change in intensity of the SERS signal, we carry out Fourier transform infrared (FTIR) spectroscopy analyses of the samples to observe if there is any chemical bonding present at day 28. Figure 3 shows the FTIR spectra of Ag and Au nanorods at day 28. It is noted, but not shown here, that there are no detectible peaks in the FTIR spectra on day 1 for either Ag or Au. In the 28 day data a strong absorption peak is observed at ~550 cm^−1^ which corresponds to bonding between Ag and S^9^. In comparison, there is no detectable attachments to Au nanorods in this binding region, and the SERS sensitivity remains constant over time. Further, absorption peaks are observed at ~2850 cm^−1^ and 2920 cm^−1^ on the Ag nanorods, and are characteristic of hydrocarbons being present on the surfaces^11^. This comparison further supports that the correlation of S attachment, as well as possible hydrocarbon fouling, and not morphological change, are responsible for a major reduction in SERS signal for Ag nanorods stored in air over the course of weeks^12^. It is noted that peaks indicating the formation of surface oxide on the Ag nanorods are not visible in the FTIR spectra at 21 or 28 days, but are visible at day 115. As the significant change in SERS intensity is observed before the formation of surface oxide, the attachment of S and hydrocarbons to the surfaces of the Ag nanorods dominates over the formation of oxide. The substantial damping of the SERS signal caused by AgS exceeds that of surface oxidation and morphological change, and the ensuing effects are not discernable here.

## Discussion and Conclusions

Before concluding, we briefly discuss the implications of our results to the SERS applications of Ag and Au nanorods. Even after chemical contamination, we observe a strong SERS enhancement by the Ag nanorods out to four months (115 days) and superiority of signal-to-background ratio of Ag nanorods over Au nanorods. It is a logical extension that Ag nanorods may be conditioned by storage in air for 21 or more days prior to use if stability and comparability of the Raman signal over several days or weeks is desired. Our results, and results in the literature^13^, demonstrate that the SERS signal is unstable over the first 21 days, which is not ideal for cross-comparison between different samples on different days, and conditioned samples may perform better in the scenario where gross signal enhancement is not the most important characteristic.

To summarize, this paper reports on the initial degradation, and eventual stabilization, of SERS signal intensity of Ag nanorods stored in air over four months. As shown via SEM, Ag nanorod morphology remains largely unchanged over 28 days, although the SERS signal intensity drops by more than 20% in the same time period. FTIR spectroscopy of the samples over the same time period shows that Ag nanorods surfaces demonstrate signs of S and hydrocarbons, which correlate to the drop in SERS intensity. As an inert analog, Au nanorods tested under the same conditions show no surface signals of S or hydrocarbons and have a very stable, but weaker, SERS intensity over four months. These results suggest that after an initial conditioning period of approximately 21 days, Ag nanorods have very stable SERS enhancement and may be used for comparative tests.

## Methods

Ag and Au nanorods are grown using electron beam PVD onto ultrasonically cleaned <100> silicon (Si) wafers with native oxide^14^. First, the wafers are ultrasonically cleaned with acetone, ethanol, and de-ionized water and are then allowed to naturally dry in air. The wafers are then mounted at the top of the vacuum chamber, which is a stainless steel tank with a source to substrate throw distance of approximately 36 cm, onto precision machined holders, which maintain the normal of the wafer at 87° relative to the source normal. Ag and Au source materials, which are 99.99% pure (Kurt J. Lesker Co.) ..., are placed into crucibles in the electron beam source at the bottom of the chamber. The vacuum chamber is then evacuated down to a base pressure of 1 × 10^−5^ Pa and deposition takes place at a working pressure of better than 1 × 10^−3^ Pa. Deposition is monitored with a quartz crystal microbalance, which is located next to the wafers at perpendicular incidence to the source normal, and the deposition rates for both Ag and Au are controlled to 1.0 nm/s with total film thicknesses of 750 nm. Immediately after growth, Ag and Au nanorods are imaged using a Hitachi S-4800 field emission scanning electron microscope at an acceleration voltage of 10.0 keV and working distance of 8 mm. FTIR spectroscopy is then performed on a Perkin Elmer Spectrum Two integrating from 500 cm^−1^ to 3500 cm^−1^ for 60 seconds. Raman spectroscopy is performed by detecting aqueous 1 × 10^−6^ M Rhodamine 6 G (R6G) (Sigma Aldrich Co.) using a Horiba T64000 Raman Spectrometer equipped with the microscope utility and liquid nitrogen cooled CCD detector. The samples are submerged in an aqueous solution for 1 hour, then removed and rinsed once in de-ionized water and allowed to dry in air. The 10 X Olympus objective focuses the 532 nm excitation laser to a ~10 μm spot on the surface of the sample. The laser power does not exceed 10 μW. We use the spectrometer in the “direct mode” by bypassing the pre-monochromator and sending the scattered light directly to the spectrograph equipped with a 600 lines/mm holographic grating. Kaiser Optics super notch filter supresses the intensity of the laser line. The entrance slit of the spectrometer is set at 200 μm. The accumulation time is set at 1 second with 25 accumulations per spectrum. The spectral range extends from 250 to 2250 wavenumbers. 10 scans are taken for each measurement, and the raw data are then averaged and standard deviations for each wavenumber are calculated. Signal-to-background ratio is calculated by comparing the averaged raw value of the R6G peak at 1645 cm^−1^ to background value at 1700 cm^−1^. The samples for days 1, 7, 14, 21, 28, and 115 are 0.5 cm × 0.5 cm pieces taken from the center 3 cm × 3 cm of the wafer to assure uniformity of the initial nanorod structure. Between days 1 and 115, samples are stored in petri dishes in a closed dark cabinet held in a climate controlled room with ambient temperature of ~23 °C.
